# Human Osteochondral Explants as an Ex Vivo Model of Osteoarthritis for the Assessment of a Novel Class of Orthobiologics

**DOI:** 10.3390/pharmaceutics14061231

**Published:** 2022-06-10

**Authors:** Chiara Giannasi, Laura Mangiavini, Stefania Niada, Andrea Colombo, Elena Della Morte, Valeria Vismara, Andrea Ambrosanio, Paolo Savadori, Sara Casati, Giuseppe M. Peretti, Anna Teresa Brini

**Affiliations:** 1 Department of Biomedical Surgical and Dental Sciences, University of Milan, 20129 Milan, Italy; paolo.savadori@unimi.it (P.S.); sara.casati@unimi.it (S.C.); anna.brini@unimi.it (A.T.B.); 2 IRCCS Istituto Ortopedico Galeazzi, 20161 Milan, Italy; laura.mangiavini@unimi.it (L.M.); stefania.niada@grupposandonato.it (S.N.); elena.dellamorte@grupposandonato.it (E.D.M.); giuseppe.peretti@unimi.it (G.M.P.); 3 Department of Biomedical Sciences for Health, University of Milan, 20129 Milan, Italy; 4 Residency Program in Orthopedics and Traumatology, University of Milan, 20129 Milan, Italy; a.colo91@gmail.com (A.C.); vale.vismara@gmail.com (V.V.); andrea.ambrosanio@gmail.com (A.A.)

**Keywords:** secretome, conditioned medium, extracellular vesicle, mesenchymal cell, osteoarthritis, osteochondral explant, orthobiologic

## Abstract

Osteoarthritis (OA) is a highly prevalent joint disease still lacking effective treatments. Its multifactorial etiology hampers the development of relevant preclinical models to evaluate innovative therapeutic solutions. In the last decade, the potential of Mesenchymal Stem Cell (MSC) secretome, or conditioned medium (CM), has emerged as an alternative to cell therapy. Here, we investigated the effects of the CM from adipose MSCs (ASCs), accounting for both soluble factors and extracellular vesicles, on human osteochondral explants. Biopsies, isolated from total knee replacement surgery, were cultured without additional treatment or with the CM from 10^6^ ASCs, both in the absence and in the presence of 10 ng/mL TNFα. Tissue viability and several OA-related hallmarks were monitored at 1, 3 and 6 days. Specimen viability was maintained over culture. After 3 days, TNFα induced the enhancement of matrix metalloproteinase activity and glycosaminoglycan release, both efficiently counteracted by CM. The screening of inflammatory lipids, proteases and cytokines outlined interesting modulations, driving the attention to new players in the OA process. Here, we confirmed the promising beneficial action of ASC secretome in the OA context and profiled several bioactive factors involved in its progression, in the perspective of accelerating an answer to its unmet clinical needs.

## 1. Introduction

Osteoarthritis (OA) is an age-related, degenerative joint disease affecting millions of people worldwide and causing important social and healthcare burdens [1]. Once considered a disease of the articular cartilage, now the contribution of the underlying tissues has been acknowledged [2]. Indeed, physiologically, the subchondral bone together with the articular and the calcified cartilage form a biocomposite—the osteochondral unit—specialized in supporting loads and allowing movement. During OA evolution, the functional and structural properties of these tissues undergo marked alterations. The pathological process counts several hallmarks, starting from the hypertrophic shift of articular chondrocytes, whose enhanced proliferation and altered gene expression lead to cartilage degradation [3]. Over time, new bone formation at the joint margins and inflammation occur. OA symptoms such as pain and stiffness can be managed pharmacologically, but to date the tissue damage cannot be inhibited nor reverted [4]. Moreover, the most conservative surgical approaches, such as microfracture, drilling and mosaicplasty, lead to the formation of low-quality fibrocartilage [5]. Ultimately, arthroplasty remains the only choice for joint functional restoration. Therefore, the unmet needs of achieving an appropriate cartilage regeneration and of developing disease-modifying, anti-OA drugs represent primary goals in orthopedic research. In the last years, a new class of substances called orthobiologics, a combination of the words orthopedics and biologics, has emerged as an innovative strategy to improve the clinical outcome in a variety of orthopedic conditions. These autologous or allogeneic products have entered the clinics, alone or in conjunction with gold standard procedures, with the aim of promoting the healing of musculoskeletal tissues, in particular tendons, ligaments, cartilage and bone [6]. A variety of preparations fall under this definition, including blood derivatives (such as platelet rich plasma), bone marrow concentrates, fat grafts or stromal vascular fraction. The therapeutic potential of the latter preparations correlates with the presence of Mesenchymal Stem/stromal Cells (MSCs), respectively, bone marrow- and adipose-tissue-derived ones (BMSCs and ASCs) [7,8]. Over the years, in vitro and in vivo studies have proven the effectiveness of MSCs in counteracting cartilage damage [9] and, to date, more than 120 clinical trials have tested their safety and efficacy against OA (source: clinicaltrials.gov). Not only do MSCs have the versatility to differentiate into cells of the mesodermal lineage (e.g., chondrocytes and osteoblasts), with clear implications in terms of tissue regeneration, but, most importantly, these cells orchestrate the local microenvironment through paracrine mechanisms. Accordingly, in recent years, scientific interest steered towards the study of MSC secretome, the conditioned medium (CM), as a promising cell-free therapeutic option. CM consists of the plethora of bioactive factors, released by MSCs during in vitro culture and able to drive regeneration. It includes both soluble mediators and extracellular vesicles (EVs) with an endosomal or a plasma membrane origin and different dimensions (small and large EVs) [10]. Thus, MSC-CM recapitulates the beneficial effects of the cells of origin reducing, at the same time, the risks associated to cell therapy, with clear advantages also in terms of feasibility and costs.

Up to now, most in vitro models of OA rely on 2D or 3D cultures of chondrocytes, either isolated from articular cartilage or derived from differentiated stem cells. In this context, we recently demonstrated the anti-hypertrophic and anticatabolic action of the CM from adipose-derived MSCs (ASCs) on articular chondrocytes grown in monolayer [11,12]. Although informative, these models fail to consider the close interplay between bone and cartilage that drives OA onset and progression. Consequently, the biological and mechanistic effects exerted by subchondral bone on articular cartilage and influencing OA dynamics are still poorly characterized. The deep investigation of the crosstalk between these tissues is critical for a better understanding of OA pathophysiology in the perspective of developing new diagnostic tools and more effective therapeutic strategies.

With these premises, our strategy harnesses the potential of the human osteochondral explant model in mimicking the environment of OA-affected joints to assess the effects of ASC secretome (ASC-CM) in an experimental model as close as possible to the physiological situation. Here, we provide an insight on the modulation of several bioactive factors involved in OA pathogenesis, including catabolic mediators, lipids, proteases and cytokines, in order to better characterize this novel ex vivo model and to evaluate the action of ASC-CM as a potential cell-free orthobiologic.

## 2. Materials and Methods

Unless otherwise stated, reagents were provided by Sigma-Aldrich (St. Louis, MO, USA).

### 2.1. Collection and Characterization of Cell Secretome

The 10 employed CM samples derive from 7 ASC populations at IV-VII culture passage, harvested from 5 female and 2 male donors, mean age 48 y/o, age range 22–70 y/o, upon institutional board approval and written informed consent. Cell secretome was obtained from subconfluent ASCs cultured for 72 h under serum deprivation, following standard procedures [13]. Briefly, the conditioned medium was collected and centrifuged at 2500× *g*, 4 °C, to remove dead cells and debris. Then, it was concentrated using 3 kDa molecular weight cut-off filter devices (Millipore, Burlington, MA, USA), aliquoted and stored at −80 °C. This protocol for CM production allows one to preserve both soluble factors and EVs. Product characterization was performed as follows: (i) quantification of total protein content by Bradford Protein Assay (Bio-Rad, Milan, Italy), (ii) Nanoparticle Tracking Analysis (NTA) by NanoSight NS300 (Malvern PANalytical, Salisbury, UK) [14], (iii) qualitative assessment of EV morphology by Transmission Electron Microscopy (TEM) at Unitech NOLIMITS facility, University of Milan and (iv) Western Blot analysis for the expression of the typical negative (Calnexin) and positive (HSP70, FLOT1, TSG101 and CD9) EV markers, following the procedure exhaustively described in [12,15]. Moreover, 3 of the 10 ASC-CM batches employed in this study were extensively characterized for the levels of 200 cytokines, chemokines, receptors, inflammatory mediators and growth factors, as reported in [14].

### 2.2. Ex Vivo Culture of Human Osteochondral Explants

**Figure 1:**
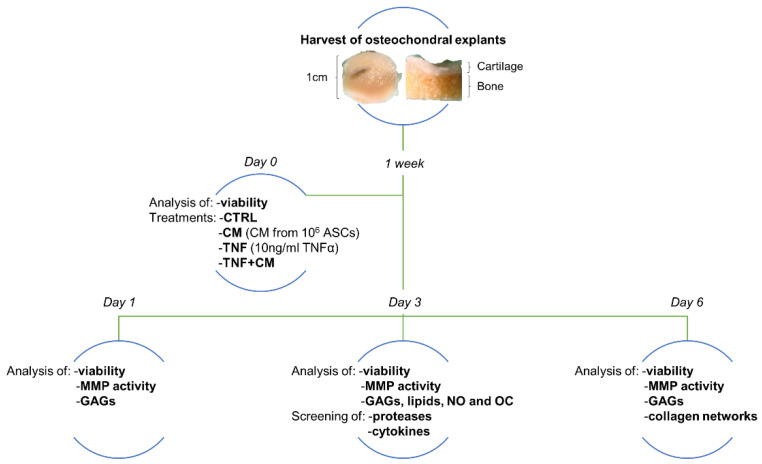
Schematic representation of the experimental setting. Osteochondral explants, collected by orthopedic surgeons, were selected based on cartilage quality, weighed and kept in culture for 1 week. After this adjustment period, tissue viability was assessed and specimens were incubated with ASC secretome derived from 10^6^ cells (CM) or challenged with 10 ng/mL TNFα, alone (TNF) or in combination with ASC-CM (TNF + CM). Untreated specimens were included as the control group (CTRL). After 1, 3 or 6 days without any further media change, viability was assessed again. At all endpoints, culture supernatants were tested for matrix metalloproteinase (MMP) activity and concentration of glycosaminoglycans (GAGs). At day 3, several lipids involved in inflammation, nitric oxide (NO) and osteocalcin (OC) were quantified, together with a panel of proteases and cytokines. At day 6, the impact of the different stimuli on the quality of collagen fibers was histologically evaluated.

Human osteochondral explants were harvested from the femur of patients undergoing total knee replacement surgery at IRCCS Istituto Ortopedico Galeazzi. The collection of waste tissues was authorized by the IRCCS Ospedale San Raffaele ethics committee (Approval Number 187/int/2019) and the study was registered in ClinicalTrials.gov (Identifier NCT04223622). Written informed consent was signed by all enrolled subjects. Donor characteristics are summarized in Supplementary S1. Within 2 h from the surgery, the osteochondral plugs (having 10 mm diameter and 4–10 mm height) were manually isolated with a trephine to avoid the necrotizing action of a drill. The explants were extensively washed in a sterile phosphate buffer (PBS, Euroclone, Pero, Italy), selected on the basis of the presence of macroscopically preserved cartilage and weighed (weight range: 0.6–1.6 g). Data derived from all downstream analyses were normalized on the wet weight of osteochondral explants, in order to account for the differences in their size (reflecting the variable thickness of subchondral bone at harvest). The specimens were cultivated up to 13 days following the experimental setting shown in Figure 1. The culture medium consisted of high glucose DMEM, 10% Fetal Bovine Serum (FBS, Euroclone, Pero, Italy), 2 mM l-glutamine, 50 U/mL penicillin, 50 µg/mL streptomycin, 2.5 µg/mL Amphotericin β and 110 µg/mL Na-Pyruvate. For the first week, prior to any treatment, media were changed every other day.

### 2.3. Viability

Prior to any treatment, each osteochondral explant was tested for viability by AlamarBlue assay (Thermo Fisher Scientific, Waltham, MA, USA). In total, 10% AlamarBlue reagent was added to the culture medium for 3 h at 37 °C in the dark. An aliquot of the solution (100 µL/explant) was then transferred in triplicate into black-bottom 96 well plates, and fluorescence (excitation λ = 530 nm, emission λ = 590 nm) was read with a Wallac Victor II microplate reader (Perkin Elmer, Milan, Italy). Explants were then washed with PBS and treated. The same analysis was performed also at day 1, 3 or 6.

### 2.4. Treatments

Selected osteochondral explants were treated as follows:

1. No treatment = CTRL
2. ASC-CM deriving from 10^6^ cells = CM
3. 10 ng/mL TNFα = TNF
4. 10 ng/mL TNFα + ASC-CM deriving from 10^6^ cells = TNF + CM

To minimize the interference of bovine contaminants in the downstream analyses, all treatments were performed in a complete culture medium with a lower percentage of animal serum (from 10% to 1% FBS) [11,12] and no further media changes occurred during culture.

### 2.5. Analyses of Catabolic and OA-Related Markers

The culture supernatants of osteochondral explants were collected and centrifuged for 5 min at 2000× *g*, 4 °C. Samples were then stored at −20 °C for further analyses. At all endpoints, matrix metalloproteinase (MMP) activity and the release of glycosaminoglycans (GAGs) were investigated. MMP activity was determined with SensoLyte 520 Generic MMP Activity Kit (AnaSpec, Fremont, CA, USA). The activation of proenzymes was obtained after incubation with 1 mM APMA (4-aminophenyl mercuric acetate) for 40 min at 37 °C, in order to preferentially assess the activity of MMP13, one of the main players in OA. After incubation with MMP substrate for 45 min, fluorescence signals (excitation λ = 490 nm, emission λ = 520 nm) were read with a Wallac Victor II microplate reader (Perkin Elmer, Milan, Italy). The catabolic release of GAGs was measured with the photometric dimethylene blue dye method [16]. A calibration curve obtained with chondroitin sulphate was used as the reference standard, and absorbance at 500 nm was measured with a Wallac Victor II microplate reader.

At day 3, culture supernatants were also tested for the content of several bioactive lipids, nitric oxide (NO) and osteocalcin (OC). Lipid quantification was performed using a QTRAP 5500 triple quadrupole linear ion trap mass spectrometer (Sciex, Darmstadt, Germany) coupled with an Agilent 1200 Infinity pump ultrahigh pressure liquid chromatography (UHPLC) system (Agilent Technologies, Palo Alto, CA, USA), following recently validated methods [17]. Briefly, 500 µL samples were extracted following a multistep procedure, then injected into the UHPLC/MS-MS system for the determination of 32 lipid species belonging to polyunsaturated fatty acids, eicosanoids, endocannabinoids and N-acylethanolamines. NO was measured using an improved Griess method based on the reduction of nitrates to nitrites (ab272517, Abcam, Cambridge, UK). Absorbance at 540 nm was measured with a Wallac Victor II microplate reader and NO concentration (µM) was inferred from a nitrite standard curve (0–200 μM). The quantification of Osteocalcin (OC) was performed with the Human Osteocalcin SimpleStep ELISA^®^ Kit (ab270202, Abcam, Cambridge, UK). Briefly, 1:25 diluted samples were tested following standard procedures and absorbance was read at 450 nm using a Wallac Victor II microplate reader. Obtained values were then interpolated with a recombinant protein standard curve to derive OC concentration (ng/mL).

### 2.6. Screening of Proteases and Cytokines

The modulation of key catabolic and inflammatory factors in selected supernatants was screened by The Proteome Profiler Human Protease Array Kit and the Proteome Profiler Human XL Cytokine Array Kit (ARY021 and A RY022B, R&D Systems, Minneapolis, MN, USA). For each group, a pool of 3 culture supernatants deriving from different experimental sets with an endpoint at day 3 was run (160 µL/set, 480 µL/array). After an overnight incubation with the samples, captured proteins were detected with biotinylated antibodies and visualized by chemiluminescent detection reagents using the ChemiDoc Imaging System (Bio-Rad, Milan, Italy). Signals were then quantified through Image Lab Software (Bio-Rad, Milan, Italy). The validation of selected cytokines was performed using the Human Magnetic Luminex Screening Assay Rk4yTGNI (R&D Systems, Minneapolis, MN, USA) following standard procedures. Briefly, the kit was customized to quantify the levels of MCP-1, IL-6, Lipocalin-2, Dkk-1 and PDGF-AA. Duplicates of each sample were tested either undiluted or 1 to 50 diluted and read through a Bio-Plex Multiplex System (Bio-Rad, Milan, Italy). Data analysis was performed with MAGPIX xPONENT 4.2 software (Luminex Corporation, Austin, TX, USA).

### 2.7. Histology

Osteochondral explants were fixed for one week in 10% neutral buffered formalin. Decalcification was obtained through 14% disodium EDTA at pH 7.2, and its endpoint was identified through the physical method [18]. Samples were then dehydrated with increasing concentrations of ethanol (from 70% to 100%) and cleared with 100% xylene (Carlo Erba reagents, Cornaredo, Milan, Italy). Finally, the specimens were embedded in paraplast paraffin (VWR, Radnor, PA, USA), and 5 µm slices were cut with a rotary microtome (Leica RM2245, Leica Microsystems, Wetzlar, Germany) and placed on super frost slides (VWR, Radnor, PA, USA). Samples were stained and analyzed following the picrosirius–polarization method [19]. First, sections were deparaffinized in xylene, then rehydrated through a sequence of ethanol solutions from 100% to 70%, ending in distilled water. Next, slices were put for 1 h in a solution of 1% Direct Red 80 (Alfa Aesar, Ward Hill, MS, USA) in saturated aqueous picric acid. After staining with picrosirius red, the excess dye was removed with two washes in 0.5% aqueous acetic acid solution for 2–3 min each. Finally, samples were dehydrated with 70% to 100% ethanol solutions, cleared with xylene and mounted with an organic mounting medium (Bio Mount HM, Bio-Optica, Milan, Italy). Slides were analyzed with an Olympus CX43 microscope coupled with a LC30 camera and two polarizers (U-POT paired with U-ANT, Olympus Corporation, Shinjuku-ku, Tokyo, Japan).

### 2.8. Statistics

Statistical analysis was performed by one-way or two-way analysis of variance (ANOVA) using Prism 9.2.0 (GraphPad Software, La Jolla, CA, USA). Differences were considered significant at *p* ≤ 0.05. Data obtained from more than 3 independent experiments are displayed as box and whiskers plots, where the box indicates the interquartile range (25th to 75th percentile), the horizontal line indicates the median and the whiskers indicate the minimum and maximum values. Data derived from 3 independent experiments are plotted as bar graphs showing the mean and the standard deviation. The clustering of protease and cytokine data was performed using ClustVis (https://biit.cs.ut.ee/clustvis (accessed on 1 April 2022)).

## 3. Results

### 3.1. Characterization of ASC-CM as A Putative Orthobiologic

**Figure 2:**
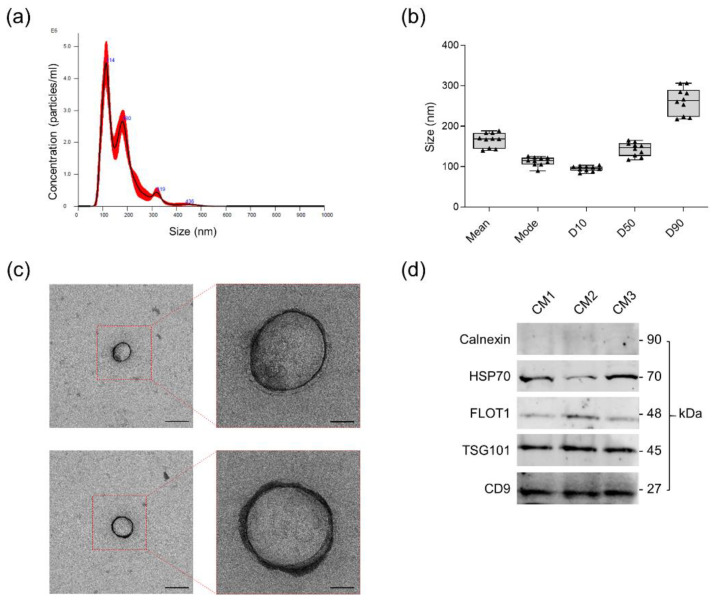
Dimensional and morphological characterization of the extracellular vesicles (EVs) contained in ASC-CM. (**a**) Representative NTA of ASC-CM. (**b**) EV size distribution in ASC-CM samples (*n* = 10). Mean, mode, D10, D50 and D90 values are reported. (**c**) Representative transmission electron microscopy (TEM) images of EVs in ASC-CM. Scale bars represent 200 nm (left panels) and 50 nm (right panels). (**d**) Expression of the typical negative (Calnexin) and positive (HSP70, FLOT1, TSG101 and CD9) EV markers in three representative ASC-CM samples by Western Blot.

Prior to use, ASC-CM batches were characterized for total protein content and vesicular composition. The mean total protein content in the CM from 10^6^ ASCs was 53.2 ± 21.9 µg, including both soluble and vesicle-conveyed proteins. Indeed, the protocol for CM production allows the retention and preserves the integrity of its vesicular fraction. Nanoparticle Tracking Analysis demonstrated a homogeneous size distribution among CM batches and indicated 100 nm as the diameter of the most represented EV population (mode = 113.0 ± 10.8 nm) (Figure 2a,b). Therefore, according to MISEV2018 nomenclature [10], most events fall within the size range of small EVs. Moreover, after a normalization with respect to the number of donor cells, a mean concentration of 9.5 ± 5.9 × 10^8^ particles/10^6^ ASCs was inferred. Transmission electron microscopy distinguished, within CM preparations, intact, single EVs with spheroid morphology, having dimensions consistent with NTA measurements (Figure 2c). At last, the expression of the canonical vesicular markers Calnexin, HSP70, FLOT1, TSG101 and CD9 in ASC-CM samples was validated by Western Blot (Figure 2d).

### 3.2. Osteochondral Explant Viability through Time

**Figure 3:**
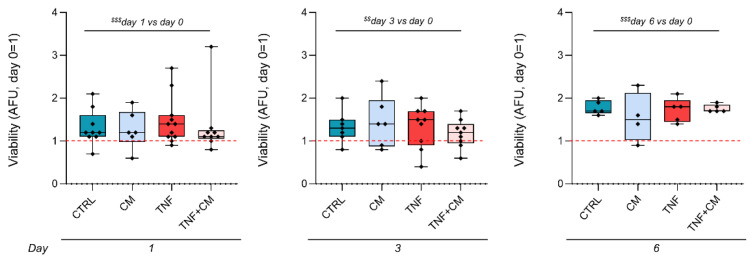
Kinetics of explant viability through time. Data are normalized on the wet weight of each explant. For each dataset, the analysis was performed at day 0, prior to any treatment, and at the different endpoints (either at day 1, 3 or 6). Data are expressed as Arbitrary Fluorescence Units (AFU) and presented as a ratio on the values obtained at day 0 (day 0 = 1, red dotted line). Significance versus day 0, obtained by two-way analysis of variance, is shown as ^$$^ *p* < 0.01 and ^$$$^ *p* < 0.001.

The viability of the osteochondral explants was determined before the treatments and at the three endpoints, confirming that tissues preserve their stability and metabolism during culture (Figure 3). Moreover, at all time points, a statistically significant enhancement of tissue metabolic activity was observed in comparison to the basal levels at day 0 (^$$$^ *p* < 0.001 for day 1 versus day 0 and day 6 versus day 0, ^$$^ *p* < 0.01 for day 3 versus day 0), indicating an increasing trend over time. Interestingly, TNFα and/or CM treatment did not interfere with explant viability.

### 3.3. MMP Activity and GAG Release through Time

**Figure 4:**
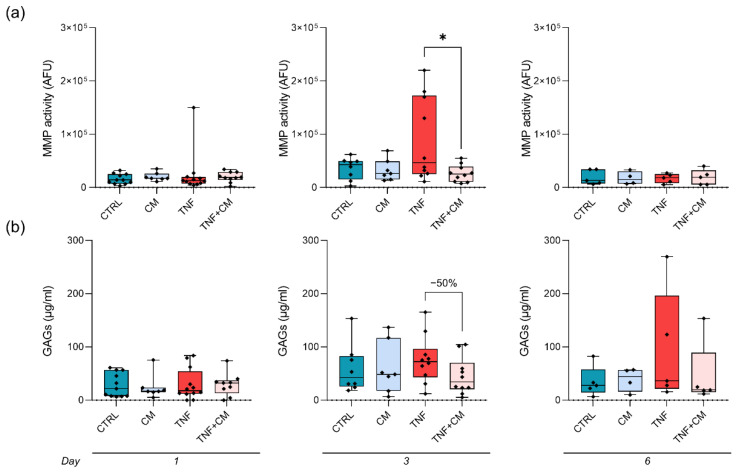
MMP activity and GAG release through time by osteochondral explants. Data are normalized on the wet weight of each explant. (**a**) Matrix metalloproteinase (MMP) activity analyzed in culture supernatants after either 1, 3 or 6 days (left to right). Data are expressed as AFU and statistical significance is shown as * *p* < 0.05. (**b**) Release of glycosaminoglycans (GAGs) at either day 1, 3 or 6 (left to right).

Since MMPs play a major role in the degenerative changes occurring during OA, we analyzed their activity. No effect of CM on unstimulated explants was ever noticed (Figure 4a). At day 3, MMP activity was induced by TNFα (+60% of CTRL), whereas CM coadministration significantly downmodulated it by 70% (Figure 4a, middle panel). Consistently, since the action of proteolytic enzymes affects the release of matrix components, the higher concentration of GAGs in the culture medium was detected at day 3 (Figure 4b, middle panel). Indeed, TNFα enhanced GAG release by 31%, and CM efficiently blunted TNFα action by restoring GAG concentration to basal levels (−50% of TNF).

### 3.4. Quantification of Bioactive Lipids, NO and OC at Day 3

**Figure 5:**
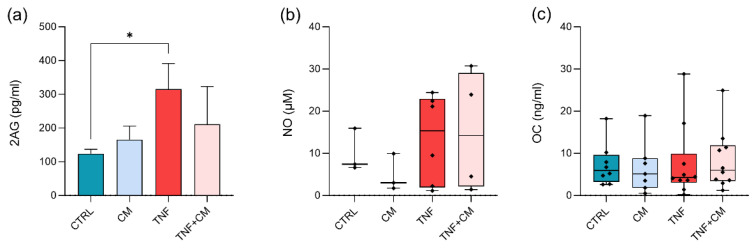
Analysis of osteoarthritis-related markers in culture supernatants at day 3. Data are normalized on the wet weight of each explant. (**a**) Quantification of 2-arachidonoilglycerol (2AG), expressed as pg/mL. Statistical significance is shown as * *p* < 0.05. (**b**) Nitric oxide (NO) production by osteochondral explants expressed as µM. (**c**) Release of Osteocalcin (OC) expressed as ng/mL.

Considering the results obtained for MMP activity and GAG release, further analyses were performed at day 3. At first, lipids involved in inflammation were searched by mass spectrometry. In total, 11 out of 32 selected lipids were quantified in the culture supernatants of the osteochondral plugs derived from three different donors (Supplementary S2). Interestingly, TNFα induced a significant upregulation of 2AG (Figure 5a, +154% of CTRL) while the coadministration of CM partly reverted it (−33% of TNF). A similar trend was also observed for AA and DHA (Supplementary S2). Conversely, the prostaglandins PGD2, PGE2 and PGF2 were strongly downmodulated by TNFα (decrease of at least −60% compared to CTRL, Supplementary S2). The production of nitric oxide (NO), a catabolic mediator involved in the inflammatory process, was also analyzed. NO quantification was unreliable in most CTRL and CM explants, while TNFα strongly enhanced its production independently of the presence of CM (Figure 5b). At last, the levels of osteocalcin (OC), a marker of OA-related impairment in cartilage and bone [20], were investigated. Unexpectedly, OC release by the osteochondral tissues was stable following all treatments (Figure 5c).

### 3.5. Profiling of Proteases and Cytokines at Day 3

**Figure 6:**
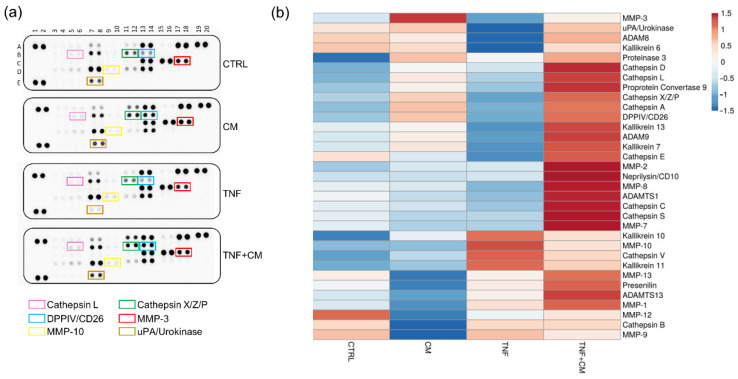
Profiling of proteases released by osteochondral explant at day 3. (**a**) Protease arrays comparing the supernatants of osteochondral explants cultured for 3 days under different stimuli. Selected proteins are highlighted by rectangles: Cathepsin L (pink), Cathepsin X/Z/P (green), DDPIV/CD26 (blue), MMP-3 (red), MMP-10 (yellow) and uPA/Urokinase (brown). (**b**) Heatmap showing the differential expression of the detected proteases among groups.

To better characterize our experimental model and to unravel possible treatment-dependent modulations, the supernatants of osteochondral explants were screened for a broad panel of proteases and cytokines. Due to the nature of the samples, 33 of the 35 tested proteases were always detectable, suggesting that osteochondral explants express high levels of degrading enzymes (Figure 6a,b, Supplementary S3). TNFα treatment upregulated MMP-10 expression, whereas CM downmodulated it, validating our data on generic MMP activity (Figure 4a). Several proteases, among which are Cathepsin L and Cathepsin X/Z/P, DPPIV/CD26 and MMP-3, were enriched in CM-treated groups, confirming their presence in ASC secretome [11,21]. Of note, TNFα reduced the expression of uPA/Urokinase, and the coadministration of CM restored it (Figure 6a,b).

**Figure 7:**
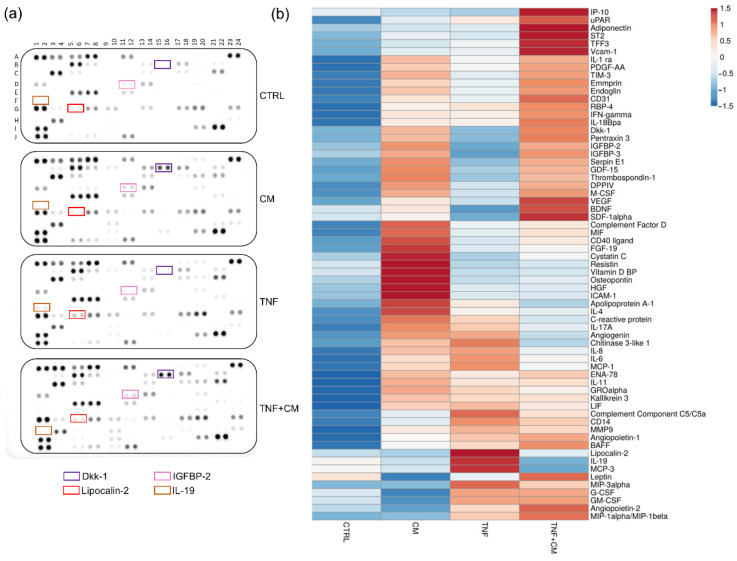
Profiling of cytokines released by osteochondral explants at day 3. (**a**) Cytokine arrays in supernatants of osteochondral explants cultured for 3 days under different stimuli. Selected proteins are highlighted by rectangles: Dkk-1 (purple), IGFBP-2 (pink), Lipocalin-2 (red) and IL-19 (brown). (**b**) Heatmap showing the differential expression of the detected cytokines among groups.

In total, 65 of the 105 cytokines tested by a proteome profiler were reliably quantified in all the supernatants (Figure 7a,b, Supplementary S4). Out of these, several molecules were upregulated by TNFα treatment and for some of them (e.g., MIP-3α, IL-6 and IL-19) CM reverted this upregulation to different extents. Furthermore, multiple factors resulted/were enriched in the secretome-treated groups, suggesting their presence in the CM rather than an active release by the osteochondral tissues. Consistently, 21 molecules, among which are Dkk-1 and IGFBP-2, were recently quantified in several CM batches by our laboratory [14]. The levels of selected molecules were validated by multiplex assay, as shown in Supplementary S5.

### 3.6. Analysis of Collagen Networks

**Figure 8:**
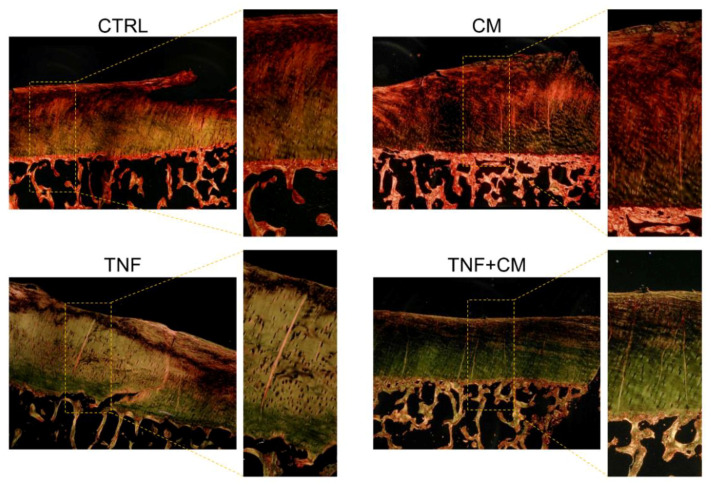
Representative images of histological sections analyzed with the picrosirius–polarization method. Osteochondral explants were fixed after 6 days of treatments. For each group, images were acquired at 20× and 40× magnification.

Knee OA is often associated with an impairment of collagen networks. The structure and spatial organization of the collagen matrix in the cartilage of osteochondral specimens were analyzed through the picrosirius–polarization method. This procedure qualitatively discriminates fibers based on their diameter, as thin bundles are visualized in green and thicker ones in yellow-red [19]. As shown in Figure 8, both the CTRL and CM groups presented thin collagen fibers in the area close to the trabecular bone, and thicker ones in the superficial margin. This fiber pattern was recognizable and regular throughout all of the specimens. Interestingly, in samples treated with TNFα, independently from CM, fibers lost this clear reticular pattern and arranged in a less organized manner, with their color turning almost entirely to green. The evidence of an impairment in cartilage quality following TNFα stimulation is also supported by Safranin O staining for proteoglycan content (Supplementary S6).

## 4. Discussion

OA progression entails marked alterations in the composition, structure and function of all the articular tissues. Given OA’s high incidence and the lack of effective therapeutic options, the need for relevant experimental models to disclose its etiology and screen new candidate drugs is a hot topic in orthopedics. Here, we propose an ex vivo OA model based on the culture of human osteochondral explants, either unstimulated or challenged with TNFα, to evaluate a new therapeutic strategy for OA. Compared to the classical in vitro settings, this model presents several advantages: (i) it allows one to examine chondrocyte behavior in the physiological context, i.e., in the presence of an extracellular matrix, thus avoiding the dedifferentiation that often occurs in monolayer cultures [22,23]; (ii) it enables one to evaluate the influence of intertissue crosstalk, a crucial aspect of OA progression; and (iii) it is compliant to 3R standards, the guiding principles of reduction, refinement and replacement of in vivo experiments, as it represents a valid alternative to animal testing. OA pathophysiology is driven by cytokines, mainly IL-1β and TNFα [24]. We chose TNFα as a chemical cue to mimic OA as it is produced by synovial tissue and cartilage under pathological conditions, triggers tissue degradation, drives inflammation and causes hyperalgesia [25]. The potential of the osteochondral explant model in recapitulating the pathophysiological environment of OA-affected joints has been exploited to test the efficacy of ASC secretome as a novel candidate orthobiologic. The action of this cell-free product, or its subcomponents (EVs and soluble factors), is being extensively investigated in the treatment of OA [26].

Since a certain degree of variability has been described, besides inter-donors, also among osteochondral plugs harvested from a same joint [27], after selecting only macroscopically preserved tissues we implemented a one-week buffering culture period prior to the different treatments. We followed this strategy in order to minimize the impact of the microenvironment of origin on the ex vivo behavior of the specimens. In our setting, no correlation between donor features (Supplementary S1) and explant response to ASC-CM and/or TNFα was found. However, since in OA a point of no return for cartilage recovery has been described [28], the predictive role of patient characteristics on the degree of responsiveness to the treatments cannot be excluded. In the future, the evaluation of the effects of the different stimuli on specimens from a larger patient cohort may clarify this aspect. At first, we confirmed a maintained explant viability and even a significantly increased metabolism during culture independently from the cytokine and/or secretome stimulation. Our results at day 3 show a significant blunting of TNFα-induced MMP activity by ASC-CM, which is consistent with our previous reports on chondrocyte monolayers [11,12]. We ascribe this important action mainly to the abundance in the CM of Tissue Inhibitors of MMPs (TIMPs), as previously reported [11,13,21,29,30]. These regulators can be both conveyed through EVs and released as soluble factors [12,31]. The complete retaining of ASC-EVs throughout the concentration process of ASC-CM allows the use of a complete product enriched in both vesicular and soluble factors. This aspect is particularly relevant considering that EVs shuttle miRNAs that are involved in a variety of pathways including inflammation and regulation of OA [32,33]. Beyond TIMPs, the contribution of bioactive factors of a different nature in the reduction of MMP activity can also be hypothesized: indeed, the presence in ASC-EVs of miRNAs known to inhibit the expression of MMPs, among which are hsa-miR-127–3p and hsa-miR148a [34,35], has been recently demonstrated [32]. In cartilage, the action of MMPs and other proteolytic enzymes, such as hyaluronidases and aggrecanases, drives the degradation and release of matrix components [36]. As a hallmark of extracellular matrix deterioration, GAG loss was measured over time [37]. Consistently with the results on MMP activity, the peak of cartilage breakdown was detected at day 3, when the beneficial action of ASC secretome was also evident. At the same time point, we investigated lipid profile, NO production and OC release. The analysis of bioactive lipids under the different stimuli discloses interesting modulations that may reflect changes in the inflammatory status of the biopsies. In detail, a significant upregulation of 2AG by TNFα and a partial counteraction by CM were detected. The endocannabinoid system regulates several OA-related pathophysiological processes, including articular metabolism, pain and pain-related emotional manifestations [38,39]. In this context, the upregulation of 2AG fits with previous data reporting its significantly higher plasma levels in OA-affected patients compared to healthy volunteers [39]. In inflamed joints, NO acts as a proinflammatory and catabolic mediator, activating MMPs, suppressing the synthesis of extracellular matrix components and promoting chondrocyte apoptosis [40]. In our ex vivo setting, NO was consistently quantified only following TNFα stimulation, in agreement with the evidence that this cytokine triggers NO production by cartilage [40]. No straightforward effect of ASC-CM was observed. Osteocalcin is a small, secreted protein classically considered a marker of mature osteoblasts [41]. Its expression in cartilage, together with other bone matrix proteins, directly correlates with OA progression and joint damage, reflecting chondrocyte differentiation and hypertrophic shift [42]. Moreover, OC levels are increased in the subchondral bone osteoblasts of OA patients [20]. In our model, no modulation of OC was detectable, neither by TNFα nor CM. On the contrary, we previously demonstrated a strong increase in OC production by TNFα-stimulated chondrocytes and a counteracting action by CM [11]. This discrepancy is most likely linked to the major contribution of the bone compartment in the current setting. In order to overcome this issue, and to better mimic the articular microenvironment where the crosstalk between bone and cartilage is restricted to the subchondral bone plate, we are currently pursuing the creation of two isolated culture chambers using custom-made 3D printed scaffolds, as recently reported by others [43,44]. The profiling data on the levels of proteases and cytokines provide an overview on interesting catabolic and inflammatory factors present in the articular microenvironment. Among others, the modulation by TNFα of urokinase plasminogen activator (uPA) and its receptor (uPAR) emerged. This system plays a central role in matrix remodeling and its unbalance correlates with different pathologies, including OA [45]. The provided clue of a TNFα-dependent strong decrease in uPA, coupled with a slight upregulation of its receptor uPAR, will deserve a deeper investigation in the future. Finally, a qualitative histological evaluation performed 6 days after the treatments suggests a negative impact of TNFα on the structure of collagen networks. This preliminary evidence requires further investigations on a larger number of samples and at different time points, in order to understand the dynamics of fiber perturbation and disclose possible counteracting effects by ASC-CM.

## 5. Conclusions

Here we confirmed a promising beneficial action of ASC secretome in counteracting OA-related hallmarks, mainly MMP activity and GAG release. Moreover, we provided a broad-spectrum analysis on mediators of different natures participating in the pathological changes affecting the osteochondral milieu. To the best of our knowledge, this is the first study describing the levels and the modulations of such a wide variety of bioactive players ex vivo on human articular biopsies. We hope that our data may open the way for a deeper investigation of both the therapeutic potential of ASC secretome (eventually optimized through the pooling of different batches in order to blunt donor-related differences) and the molecular pathways driving OA progression, in the perspective of disclosing potential therapeutic targets and developing effective strategies to overcome the limitations of the currently available treatments.

## Data Availability

Data supporting the reported results are openly available in Zenodo at https://zenodo.org/record/6623254#.YqG_mBrP1PY.

## Supplementary Materials

Reference [46] is cited in the Supplementary Materials. The following supporting information can be downloaded at: https://www.mdpi.com/article/10.3390/pharmaceutics14061231/s1, Supplementary S1: Characteristics of the donors included in the study. The table shows the number of enrolled donors for each endpoint, their sex, age, BMI and OA grade at the day of knee replacement surgery. Supplementary S2: Lipids quantified in explant supernatants at day 3. Data are normalized on the wet weight of each explant. The list of quantified bioactive lipids, together with their levels expressed as ng/mL or pg/mL, is reported. Bar graphs show the modulation of selected molecules under the different stimuli. Supplementary S3: Proteases detected in explant supernatants at day 3. The list of quantified proteases is shown together with the mean spot density expressed as arbitrary units (AU). Supplementary S4: Cytokines detected in explant supernatants at day 3. The list of quantified cytokines is shown together with the mean spot density expressed as arbitrary units (AU). The molecules previously quantified in CM batches are highlighted in blue and their mean content is reported as pg/10^6^ ASCs. Supplementary S5: Validation of selected cytokines in explant supernatants at day 3. Data are normalized on the wet weight of each explant. The levels of MCP-1, IL-6, LCN2, Dkk-1 and PDGF-AA under different stimuli are expressed as ng/mL or pg/mL. The bar graph shows the modulation of Dkk-1. Statistical significance is shown as *** *p* < 0.001. Supplementary S6: Representative images of histological sections stained with Safranin-O. Osteochondral explants were fixed after 6 days of treatments. Images were acquired at 20× magnification.
