# Supplementary material for: Human Osteochondral Explants as an Ex Vivo Model of Osteoarthritis for the Assessment of a Novel Class of Orthobiologics
Source: Pharmaceutics. 2022 Jun 10;14(6):1231. doi: 10.3390/pharmaceutics14061231 (PMC9229444; doi:10.3390/pharmaceutics14061231)
Supplement: Supplementary file 1 [file pharmaceutics-14-01231-s001.zip › Supplementary S6.pdf]

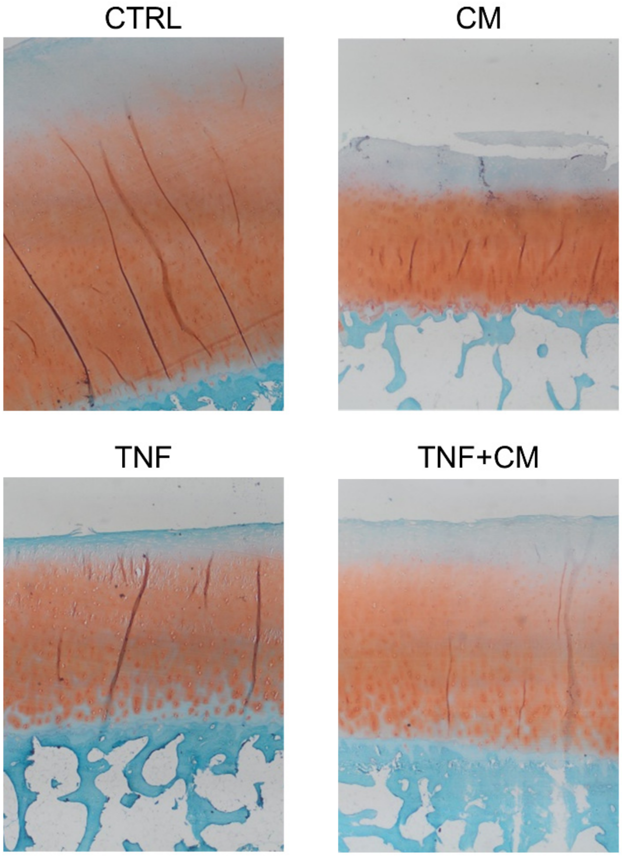

Supplementary S6. Representative images of histological sections stained with Safranin-O. Osteochondral explants were fixed after 6 days of treatments. Images were acquired at 20x magnification.

All samples show a moderate reduction of Safranin O-Fast Green staining, extending from the cartilage surface to the mid zone. This pattern is typically associated to OA-related cartilage damage [46]. TNFα-treated samples, independently from CM, show a further discoloration at the tide mark level, indicating an additional loss of proteoglycans. Taken together, these results correlate with the evidence of a TNFα-induced alteration in the organization of collagen fibers obtained through the picrosirius-polarization method (Figure 8).

Materials and Methods

Slides were deparaffinized and hydrated to distilled water following standard procedures. Samples were then stained with Weigert's iron hematoxylin solution for 10 minutes, washed in running tap water, stained with fast green solution for 5 minutes, briefly washed in 1% acetic acid solution and stained with 0.1% safranin O solution for 5 minutes. Reagents were purchased from Carlo Erba (Cornaredo, Milan, Italy). After dehydration and cleaning with xylene, slides were mounted (Bio Mount HM, Bio-Optica, Milan, Italy) and analyzed by bright-field microscopy (BX51 Olympus, Tokyo, Japan).
